# Chronic hepatitis B genotype E in African migrants: response to nucleos(t)ide treatment in real clinical practice

**DOI:** 10.1186/s12879-018-3469-y

**Published:** 2018-11-14

**Authors:** José Ángel Cuenca-Gómez, Ana Belén Lozano-Serrano, María Teresa Cabezas-Fernández, Manuel Jesús Soriano-Pérez, José Vázquez-Villegas, Matías Estévez-Escobar, Isabel Cabeza-Barrera, Joaquín Salas-Coronas

**Affiliations:** 1 Tropical Medicine Unit, Hospital de Poniente, Carretera de Almerimar s/n, PD: 07400 Almería, El Ejido Spain; 2 Tropical Medicine Unit, Distrito Poniente, Almería, Spain; 3 Digestive Service, Hospital de Poniente, Almería, El Ejido Spain

**Keywords:** Hepatitis B, Genotype E, Tenofovir, Entecavir, African migrants

## Abstract

**Background:**

Hepatitis B virus (HBV) genotype E is a poorly studied genotype that almost exclusively occurs in African people. It seems to harbour intrinsic potential oncogenic activity and virological characteristics of immune scape but a paucity of information is available on clinical and virological characteristic of HBV genotype E-infected patients as well as on the efficacy of anti-HBV drugs for such patients. The increasing flow of migrants from high endemic HBV sub-Saharan Africa, where genotype E is the predominant one, to Western countries makes improving such knowledge critical in order to deliver proper medical care.

**Methods:**

Prospective observational study of naïve patients of sub-Saharan origin treated for chronic HBV genotype E infection at a Tropical Medicine clinic sited in Spain from February 2004 to January 2018. The aim of the study was to describe the response of chronic HBV genotype E infection to nucleos(t)ide analogues (NA), entecavir or tenofovir, in real clinical practice.

**Results:**

During the study period, 2209 sub-Saharan patients were assisted at our Tropical Medicine Unit and 609 (27.6%) had chronic HBV (CHB) infection. Genotype information was available for 55 naïve patients initiating treatment with NA (entecavir or tenofovir), 43 (84.3%) of them being genotype E, although 15 were excluded because they did not meet study inclusion criteria. Thus, a total of 28 CHB genotype E patients were included and followed for 24 months at least. Twenty-one patients were in HBeAg-negative chronic hepatitis phase and 7 patients in HBeAg-positive chronic hepatitis phase. After one year of treatment, among those with good adherence, 89.4% (17/19) of the HBeAg-negative patients and 80% of the HBeAg-positive ones had undetectable viral loads. Response rates reached 100% in both groups after 15–18 months of follow-up. Out of the 7 HBeAg-positive patients, 6 (85.7%) presented HBeAg loss in a median time of 31.8 months. Neither serious adverse effects nor hepatocarcinoma cases happened during the study period.

**Conclusions:**

HBV genotype may influence disease progression and antiviral response. Our study provides precious information on the efficacy and safety of NA treatment for CHB genotype E infection, a fairly unknown genotype with and increasing epidemiological impact.

## Background

Hepatitis B virus (HBV) chronic infection is a very prevalent disease worldwide. It is estimated that more than 240 million people are infected and that around 686,000 people annually die from complications of this disease, including liver cirrhosis and hepatocarcinoma [1].The vast majority of them live in low- and middle-income regions such as sub-Saharan Africa, where roughly more than 50 million people are infected.

Hepatitis B could be considered, in some parts of the world, a neglected disease for a number of reasons [2]:the major burden of morbidity and mortality from HBV is borne by tropical and subtropical countries; HBV infection is a silent disease leading to a large pool of undiagnosed infection; it disproportionately affects populations living in poverty; it causes stigma and discrimination; lack of public/media representation because HBV is “eclipsed” by higher profile infections such as HIV or malaria; lack of existing investment and development of infrastructure through which to provide education, prevention, diagnosis, and treatment; poor-quality data contributing to misinformation about epidemiology and risk factors and lack of assessment regarding feasibility of interventions; and, finally, lack of major dedicated funding agencies. As a result, mortality due to viral hepatitis (HBV and HCV) is increasing while mortality from other diseases such as HIV and malaria has declined [3].

To date, 10 genotypes (A-J) and more than 40 sub-genotypes have been identified for HBV. The distribution of HBV genotypes differs throughout the world [4]: genotypes A and D are the most prevalent in Europe; D in Asia; B, D and G in North America; F in South America; and E in sub-Saharan Africa, comprising up to 70% of all African HBV-infected patients.

HBV genotype may influence disease progression and antiviral response [5] and there are numerous studies related to such aspects: evolution of the disease according to genotype or risk of chronicity [6–9]; percentage of HBeAg or HBsAg loss [10, 11]; HBV DNA levels [12–15]; risk of progression of liver disease [16, 17] and risk of hepatocellular carcinoma [18–20]; and response to treatment [21–25]. However, most studies are performed on genotypes A, B, C, and D.

Although it seems to harbour intrinsic potential oncogenic activity and virological characteristics of immune scape [26], there is limited literature on the natural course of HBV genotype E chronic infection, and even fewer studies addressing its response to antiviral treatment.

A search in PUBMED with the commands “hepatitis B” AND “genotype E”, without any type of filter, retrieved 141 articles. Of these, only 10 [12, 27–35] (7.1%) describe genotype E response to treatment in a variety of distinct scenarios: HIV-HBV co-infected patients [27–30], rescue after lamivudine failure [31], adefovir phase III clinical trials including a total of 6 genotype E patients [12]; response to interferon [33, 34] and a follow-up study of HBsAg decline in entecavir-responding patients [32]. Finally, one retrospective study addresses HBV genotypes E-H response to antiviral therapy in naïve mono-infected patients but includes just 6 genotype E patients treated with nucleos(t)ide analogues [35]. Current international guidelines do not consider either patients with HBV-genotype E chronic hepatitis [36].

HBV genotype E almost exclusively occurs in African people but due to the significant migratory flow from sub-Saharan countries that has occurred in Europe in recent years, an increasing number of patients with chronic HBV hepatitis are been treated and followed up [37]. In some studies, more than 80% of these patients had a genotype E [38]. A better knowledge of the evolution of CHB genotype E infection and its response to treatment is of great importance when making recommendations that could differ from those issued for other genotypes, in order to deliver proper medical care.

Our Tropical Medicine Unit (TMU) belongs to the Hospital of Poniente, sited in the province of Almeria, in Southern Spain. It serves a population of about 270,000 people, of which around 30% are immigrants, with a high proportion of people coming from West African countries. Chronic hepatitis B is one of the most prevalent infectious diseases among immigrant patients, especially among those of sub-Saharan origin [39].

The aim of this study is to analyze the response to treatment with NA (entecavir or tenofovir) in sub-Saharan patients with CHB genotype E infection in real world practice.

## Methods

### Study design

Prospective observational study intended to describe virological outcome of naïve African patients with CHB genotype E infection treated with NA in real clinical practice.

### Study population and data collection

The study was carried out at the TMU of the Hospital of Poniente from February 2004 to January 2018. The following inclusion criteria were followed: i) CHB genotype E infection; ii) absence of coinfection with human immunodeficiency, hepatitis C or hepatitis D viruses, and absence of any other liver disease, such as autoimmune hepatitis; iii) individual follow-up for at least 24 months.

Decision to start HBV treatment was based on current recommendations of the European Association for the Study of Liver on force at the time [40, 41]. The choice of starting treatment with tenofovir or entecavir was at the discretion of the physician responsible for the patient, except in the presence of impaired renal function, where entecavir was used. On the contrary, all women of reproductive age were treated with tenofovir. Treatment with INF/PEG-INF was not considered because, although the available information is truly scarce, a recent study has shown poor response to interferon-based treatment in patients with chronic HBV genotype E infection [34], and also because of a less favourable tolerability-security profile as compared to NA treatment, specially for the migrant population, often conditioned by language barrier, infectious co-morbidities and poor socio-economic status.

For each included patient, laboratory and imaging data were prospectively registered. Data were analysed anonymously.

### Laboratory data

Serum samples were evaluated for the presence of HBsAg, anti-HBc and anti-HBs. When HBsAg was detected, HBeAg and anti-HBe were determined. Patients with HBsAg and anti-HBe positivity, viral load under 2000 IU/mL, and persistently normal transaminase levels were defined as inactive chronic carriers. The remaining patients with HBsAg positivity were classified either as HBeAg-positive chronic hepatitis (HBeAg-positive, anti-HBe-negative), or HBeAg-negative chronic hepatitis (HBeAg-negative, anti-HBe-positive), following the criteria of the European Association for the Study of the Liver on force [40, 41]. In HBsAg-positive patients, the quantification of HBV-DNA was performed by real-time PCR (COBAS Amplipre / CobasTaqman-Roche Diagnostics), with a limit of detection of 10 IU/ml. HBV genotype was determined by partial amplification and sequencing of the HBsAg coding gene.

### Follow-up

Visits were scheduled every 3 months, during the first year and afterwards. Viral load was programmed to be determined at months 3, 6 and 12 months, and every 6 months from there on. The evolution of alanine aminotransferase (ALT) and HBV-DNA viral load were analysed. Normal ALT was defined as ≤30 IU/L in men and ≤ 19 IU/L in women [42]; and undetectable viral load as HBV-DNA < 10 IU/mL.

As an actual clinical practice study, determination of HBV viral load was adapted to whenever patients came to the clinic, trying to roughly follow the aforementioned scheme. For those visits with no viral load available, it was assumed that it was detectable if it was detectable at the previous and subsequent visits (± 3 months), and undetectable if it was so at the previous and subsequent ones (± 3 months).

All patients underwent a liver ultrasound before treatment and every 6 months thereafter. An ultrasound-guided liver biopsy was performed in those patients presenting any medical reason for. METAVIR classification was used for classification of liver fibrosis and activity grade [43].

To evaluate adherence to treatment, clinical interview at every scheduled visit to the clinic and verification of patients’ drug withdrawal from the hospital pharmacy were used. To assess the safety of the treatment, urine sediment and dipstick analysis, glomerular filtration and plasma phosphorus level were measured at each visit.

### Statistical analysis

A descriptive statistical analysis was performed where continuous variables were expressed as medians and interquartile ranges (IQR), and categorical variables were described using a table of frequencies and proportions. STATA version 12 was the statistical program used to analyse the data.

### Literature review

To analyse the relevance of genotype E in the literature, a bibliographic search was carried out in PUBMED using the commands “hepatitis B” AND “genotype E”, without any type of filter.

## Results

During the study period, a total of 2209 sub-Saharan patients were assisted at the TMU; 609 (27.6%) of them presented CHB infection: 370 (60.7%) were classified as inactive chronic carriers, 181 (29.7%) were in a chronic HBeAg-negative phase, and 58 (9.5%) in a chronic HBeAg-positive phase. Treatment was initiated in 72 patients; genotype information was available in 55 of them: 43 (84.3%) genotype E, 6 genotype A, and 2 genotype D. Finally, out of the 43 patients with genotype E, 15 were excluded because they did not meet the inclusion criteria of the study. Thus, a total of 28 patients were included.

### Demographic data

Out of the 28 patients, 24 (85.7%) were men and the median age was 31.5 years (IQR 8). Median length of stay in Spain was 54 months (IQR 49). The countries of origin were Mali (7 patients), Guinea-Bissau (7 patients), Senegal (5 patients), Guinea-Conakry (3 patients), Ghana (3 patients), and Gambia, Nigeria and Burkina Faso (1 patient each).

### Hepatitis B laboratory data results

Twenty-one patients were in HBeAg-negative chronic hepatitis phase and 7 patients in HBeAg-positive chronic hepatitis phase; 26 patients (92.9%) presented abnormal elevated ALT measurements before starting treatment. Median ALT in HBeAg-negative patients was 64.5 IU/L (IQR 119), and 84 IU/L (IQR 61) in HBeAg-positive patients. One hundred per cent of patients had detectable viral load at the time of treatment initiation. Median viral load at the start of treatment for HBeAg-negative patients was 5.28 logIU/mL (IQR 6.26), and 7.23 logIU/mL (IQR 5.23) for HBeAg-positive patients. Baseline characteristics at the start of treatment are described in Table 1.

**Table 1:** Baseline characteristics at the start of treatment

	HBeAg-positive	HBeAg-negative
Number of patients: N (%)	7 (25%)	21 (75%)
Age (years)^a^	24 (8)	33 (7)
Male sex: N (%)	5 (71.4%)	19 (90.5%)
Mean length of stay in Spain (months)^a^	11 (72)	69 (28)
Co-morbidities	None	Coinfection with *S. mansoni* (2 patients) Fatty liver (1 patient)
ALT (IU/L)^a^	84 (61)	64.5 (119)
AST (IU/L)^a^	62 (26)	56.5 (93)
GGT (IU/L)^a^	30 (41)	48.5 (34)
ALP (IU/L)^a^	104 (66)	92 (35)
Total bilirubin (mg/dL)^a^	0.4 (0.2)	0.66 (0.93)
Platelets × 10^3^ μL^a^	204 (97)	168.5 (111)
Prothrombin time (%)^a^	78 (18)	90.5 (20.3)
Alpha-fetoprotein (ng/mL)^a^	4.6 (3.6)	2.5 (1.4)
HBV-DNA (log IU/mL)^a^	7.23 (5.23)	5.28 (6.26)
FIB- 4 score^a^	0.8 (1.8)	1.5 (0.9)
APRI score^a^	0.9 (1.2)	1 (1.7)
Chosen treatment	Tenofovir (7 patients)	Tenofovir (17 patients) Entecavir (4 patients)

^a^Values are median and (IQR). *ALT* Alanine aminotransferase, *AST* Aspartate aminotransferase, *GGT* Gamma-glutamyl transferase, *ALP* Alkaline phosphatase. FIB-4 score: Fibrosis-4 score. APRI score: AST to Platelet Ratio Index

### Ultrasound and biopsy findings

Hepatic ultrasonography was abnormal (showing signs of chronic liver disease) in 5 patients (17.9%). Hepatic biopsy was performed in 10 patients: 3 had stage A1F1, 3 patients A1F2, 1 patient A2F1, 1 patient A2F2, 1 patient A3F2, and 1 patient A3F3, as for METAVIR classification.

### Treatment outcome and follow-up

Treatment with tenofovir 245 mg/day was initiated in 24 patients (17 HBeAg-negative and 7 HBeAg-positive), and with entecavir 0.5 mg/day in 4 patients (all of them HBeAg-negative).

During the first year of follow up, 93% of patients had good adherence, attending to scheduled visits regularly and showing good treatment compliance. This figure almost reached 100% during the second year.

**Fig. 1:**
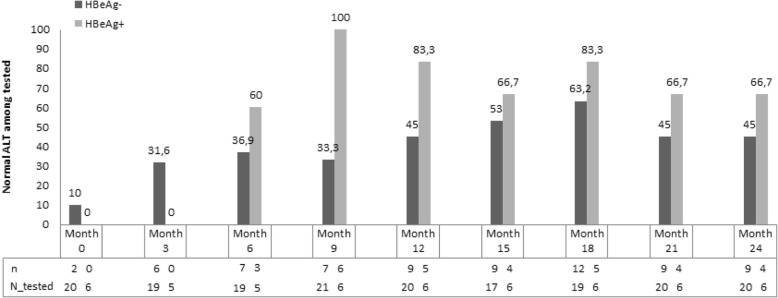
Percentage of compliant patients with normal alanine aminotranferase (ALT) levels at the different months of follow-up

**Fig. 2:**
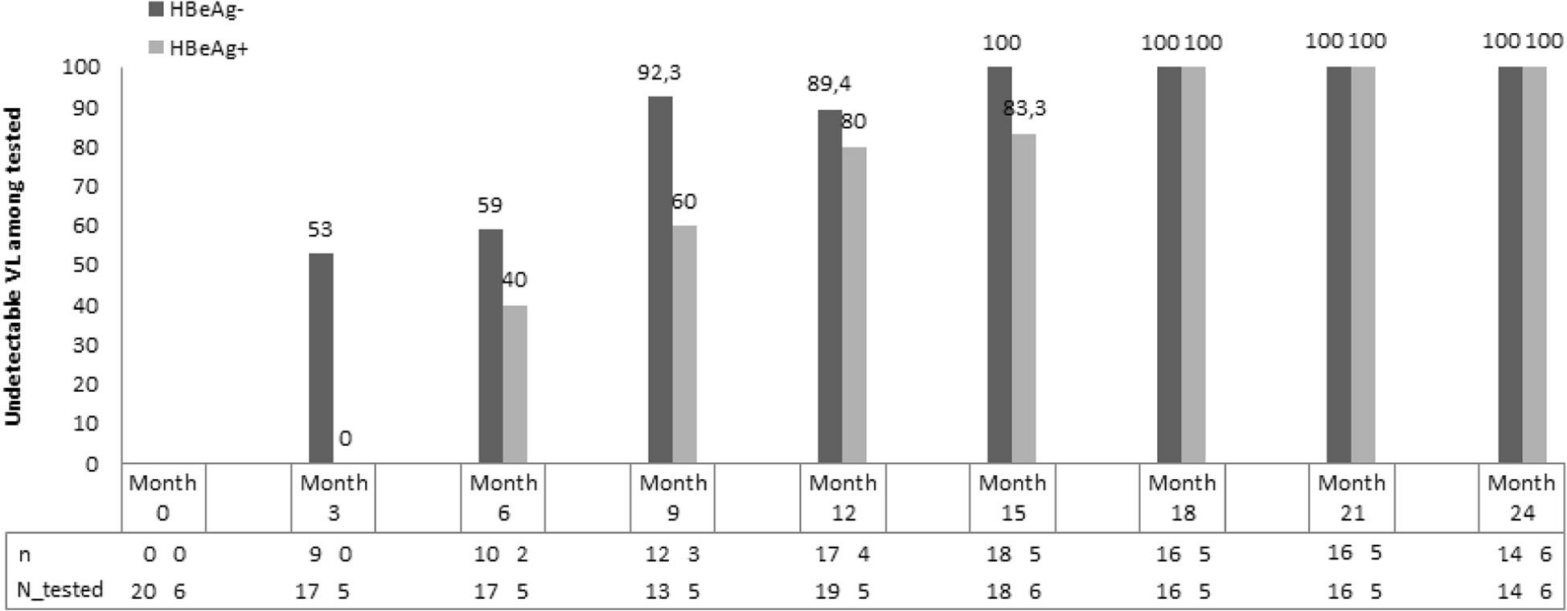
Percentage of compliant patients with undetectable viral load (VL) at the different months of follow-up

For the analysis of the evolution of ALT levels and viral load, only those patients who showed good adherence to the treatment were selected (20 HBeAg-negative patients and 6 HBeAg-positive). The proportions of compliant patients with normal ALT or undetectable viral load in each visit are shown in Figs. 1 and 2. After two years of treatment, 66.7% of HBeAg-positive patients and 45% of HBeAg-negative ones had normalized transaminases. ALT figures may have been influenced by the fact that two patients were coinfected by *S. mansoni* and one patient had a fatty liver. After one year of treatment, 89.4% (17/19) of HBeAg-negative compliant patients had an undetectable viral load, rising to 100% at month 15. In the case of HBeAg-positive compliant patients, 80% had an undetectable viral load after one year of treatment, and 100% at 18 months.

Of the 7 HBeAg-positive patients, 6 (85.7%) presented HBeAg loss (5 with anti-HBe seroconversion) in a mean time of 31.8 months. No HBsAg loss happened during follow-up in any patient. No cases of hepatocarcinoma were detected during the study period.

There were not any serious adverse reactions during the whole follow-up period. Only 6 cases of mild and self–limited hypophosphatemia were detected in tenofovir treated patients, none of them requiring discontinuation of the drug. None of the tenofovir treated patients presented evidence of impaired renal function.

## Discussion

With the data we provide, we can state that treatment with nucleos(t)ide analogues in sub-Saharan patients with CHB genotype E infection have at least as favorable results as the ones reported for other genotypes, in terms of virological response rates, normalization of transaminases, and loss of HBeAg.

These finding are relevant because sub-Saharan people are one of the populations with the highest prevalence of HBV infection of the world, and genotype E in particular is highly endemic in most of sub-Saharan Africa. Even though HBV genotype E horizontal transmission is also possible, among the migrant population coming from sub-Saharan Africa the infection is most frequently acquired vertically at birth or during the first years of life as a consequence of traditional medicine practices and tribal scarifications [44]. This fact means that, although our patients were fairly young (mean age 31.5 years), HBV infection has probably been present for all that long.

There are many studies relating to chronic hepatitis B infection in recent years but a paucity of information is available on the clinical and virological characteristics of HBV genotype E-infected patients as well as on the efficacy of anti-HBV drugs. Epidemiological studies have suggested the carcinogenic potential of genotype E, and in fact, African regions in which genotype E is endemic are characterized by a higher incidence of hepatocellular carcinoma. Although the mechanisms underlying this hypothetic oncogenic potential have not yet been clarified, they could be related to immune escape phenomena [26] as well as to other possible variables involved, such as HIV co-infection, dietary iron overload or aflatoxins consumption [45].

As there is a growing migration movement from these African regions to western countries [46], dealing with migrant patients with hepatitis B genotype E is becoming more frequent in western hepatology clinics and hospitals. Out of the actual clinical practice studies, only the studies by Marcellin [47] et al. and Boglione et al. [32, 34] include a considerable number of sub-Saharan patients, although in Marcellin’s study no genotype information is available.

HBV genotype may influence antiviral response, but the main studies that analyze the role of HBV genotype in the treatment with NA mostly deal with genotypes A, B, C and D [5, 48]. The importance of our work is that, to our knowledge, is the first prospective clinical practice study truly exploring the virological response to NA therapy in CHB genotype E patients.

There is one clinical practice study about treatment of CHB genotype E patients with NA [32], but it is focused in the decrease of HBsAg titles. In this work, the authors conclude that the decrease of HBsAg titles is smaller in the 34 patients infected by genotype E as compared to patients infected by genotypes A or D, although the analysis only included those patients who had achieved previously virological response (HBV-DNA undetectable after 24 weeks of therapy), and the meaning of HBsAg-title changes in this setting is still unclear. Another study with genotype E-infected patients, also conducted by Boglione et al. [34], explores the response to interferon therapy finding very low rates of response; 10 patients were rescued with entecavir after failing interferon treatment and virological response was achieved in 60% of them after a follow-up median of 5.2 months. There is also one retrospective study that addresses HBV genotypes E-H response to antiviral therapy in naïve mono-infected patients, but includes just 6 genotype E patients treated with nucleos(t)ide analogues [35]. Current international guidelines do not yet mention patients with HBV-genotype E chronic hepatitis [36].

Finally, a handful of articles deal with CHB genotype E patients and NA treatment in different clinical scenarios, like HIV-HBV co-infected patients [27–30], rescue after lamivudine failure [31], and the phase III studies of adefovir dipivoxil reported by Westland et al. [12] where 6 patients with genotype E were included.

In our study we have included 28 treatment-naive patients and have observed that at 12 months of treatment with NA, 89.4% of HBeAg-negative and 80% of HBeAg-positive patients negativize the viral load. This proportion increases to 100% in both groups at month 18. In other studies conducted in real clinical practice with chronic hepatitis B patients, overall viral load negativization rates at 12 months in patients treated with tenofovir ranged from 59.2 to 91.9% [24, 25, 47, 49–51]; and with entecavir, from 41 to 89.4% [21–23, 51–62]. These differences between the studies are possibly due to the diverse populations studied in each case, the different sensitivity of the PCR used to detect viral load, and to the fact that, in many studies, patients previously treated with other treatments for HBV are included. None of these studies do, however, mention HBV genotyping.

Table 2 compares those other studies that do analyze the response to treatment, as ALT normalization and viral load undetectability, according to HBV genotype. Among the few available studies, it stands out one conducted by Ono et al. [22], where genotype C predominates (70.9%), and the proportion of patients with undetectable viral load after one year of treatment is 88%, similar to that of our work. In the study of Zoutendijk [23], where genotype D predominates in non-cirrhotic patients (49%), 68% of patients have undetectable viral load after one year. Due to the relatively small number of patients included in these studies, no clear conclusions can be drawn about a faster response to treatment in patients with one genotype or another. Although these data may suggest that patients with genotypes C and E respond faster to treatment with nucleos(t)ide analogues than those with genotype D.

**Table 2:** Studies on actual clinical practice using entecavir or tenofovir where HBV genotype and virological response information is available

	No. of patients	Drug	Race	Genotype	HBeAg-negative, %	Months of follow-up (Median)	Undetectable DNA, %	ALT normal, %	AgHBe loss %
Lampertico 2011	**418**	Entecavir	–	D (90%)	83	60	100 (HBeAg+) 99 (HBeAg-)	90 (36 months)	55
Ono 2012	**474**	Entecavir	–	A (2.5%) B (14.1%) C (70.9%)	53	28	88 (12 months) 96 (48 months)	83 (12 months) 93 (48 months)	16 (12 months) 38 (48 months)
Zoutendijk 2013	**372**	Entecavir	48% White 27% Asian 25% Others	A (9.4%) B (6.7%) C (10%) D (36%)	58	20	68 (12 months) 93 (36 months)	78 (Total at the end of treatment))	17
Kim 2015	**151**	Tenofovir	100% Asian	C (100%)	39.1	13	64.2 (12 months)	97.7 (12 months)	12 (6 months) 15.2 (12 months)
Lovett 2017	**92 (55 naive)**	Tenofovir	83.7% Asian 7.6% White 4.3% African 4.4% Others	A (3.3%) B (7.6%) C (14.1%) D (3.3%)	65.5	24	59.2 (12 months) 89.3 (36 months)	86% (Total at the end of treatment)	16.7 (36 months)

Regarding normalization of transaminase levels, in the present study rates were 42.1 and 85.7% after one year in HBeAg–negative and HBeAg-positive patients respectively. These percentages reached 55.6% in HBeAg-negative and 100% in HBeAg-positive patients at 18 months of follow up. In other studies of actual clinical practice, the percentage of patients with normal transaminases at 12 months ranged from 34.6–95.5% [21–25, 47, 49–53, 56–60, 62]. These discrepant results may be explained by the different cutoff points taken for normal ALT in the different studies, and by the different stages of hepatopathy of the included patients, since in some cases they include patients with established cirrhosis and even with hepatocarcinoma.

HBeAg loss occurred in 85.7% of patients in a median time of 31.8 months. Seroconversion rate during the first year was 12.5%. Although there are few HBeAg-positive patients in our study, these results are similar to those found in other published works where these rates oscillate between 8 and 16% [22, 24, 53, 55, 61].These studies also show that HBeAg-loss rates increase significantly after more than 3 years of treatment. In these works, like in ours, no HBsAg loss is observed.

It should be noted the high adherence to treatment observed in our study: 86.7% in the first year and almost 100% in the second year. Among migrants this may be especially striking, since many are the reasons that hamper their compliance and follow-up (labour mobility, visits to their home countries, barriers to access to health care systems, etc.). The reasons for such good adherence are probably related to the collaboration of mediators and community health agents, the use of cost-free drugs, and the lack of barriers to access to the public health care system, either primary or specialized care, as is established in Andalusia, our administrative region.

The main limitation of this study is that the number of patients is not very high, although figures are according to the scarce published studies that describe the response to treatment of genotype E chronic hepatitis B patients, either with interferon or with nucleos(t)ide analogues [12, 32, 34, 35]. It would have also been interesting to compare patients with genotype E with other patients of similar origin and socioeconomic circumstances but with different genotypes. In relation to this last aspect, the number of African migrants that we deal with harbouring other genotypes is so low that does not allow valid comparisons. One last limitation is that therapeutic drug monitoring and HBsAg quantification were not available in our hospital, even though the role of the last one during therapy with oral NA is still debatable.

Nevertheless, studies like ours, that shed light on the response of CHB to different antiviral treatments depending on the genotype, are relevant. This is especially true in the case of relatively unknown genotypes like genotype E, a genotype associated to immune scape mutations [26] and whose epidemiological impact is steadily increasing in the Western world due to migration from Africa. Improving our knowledge on CHB genotype E infection and its response to treatment is critical to issue proper and specific recommendations. In addition, although access to NA in Africa is currently difficult, all evidence gathered is useful with a view to future strategies for the treatment and prevention of this disease in the countries of origin. May our work then be considered a small and humble contribution to HBV infection clinical care research.

## Conclusions

As shown in this real practice study, treatment with nucleos(t)ide analogues is safe and effective in sub-Saharan migrants with CHB genotype E infection. However, direct comparison studies seem to be necessary to conclude whether there are significant differences in the response to treatment when compared to other HBV genotypes.

Migration is altering geographical distribution of HBV genotypes around the world making considering the clinical and epidemiological implications of less known HBV genotypes, such as genotype E, critical in order to provide proper tailored care.

In our setting, where health care and antiviral drugs are cost-free and available with no barriers, high rates of adherence to treatment can be achieved. But these results could also be transferable to low- and middle-income African populations if therapy with such drugs were to be generally available in the future, contributing to research into a neglected disease that affects a large part of the population living in the poorest regions of the world.

## Abbreviations

ALP: Alkaline phosphatase
ALT: Alanine aminotransferase
anti-HBc: Hepatitis B core antibody
anti-HBe: Hepatitis B envelope antibody
anti-HBs: Hepatitis B surface antibody
AST: Aspartate aminotransferase
CHB: Chronic Hepatitis B
CI: Confidence interval
GGT: Gammaglutamyl transferase
HBeAg: Hepatitis B envelope antigen
HBsAg: Hepatitis B surface antigen
HBV: Hepatitis B virus
HCV: Hepatitis C virus
HIV: Human immunodeficiency virus
INF/PEG-INF: Interferon/pegylated-interferon
IQR: Interquartile range
NA: Nucleos(t)ide analogues
PCR: Polymerase chain reaction
SD: Standard deviation
TMU: Tropical Medicine Unit
